# Validation of the Spanish version of the Oral Health Impact Profile (OHIP-14Sp) in elderly Chileans

**DOI:** 10.1186/1472-6831-14-95

**Published:** 2014-08-04

**Authors:** Soraya León, Daniel Bravo-Cavicchioli, Gloria Correa-Beltrán, Rodrigo A Giacaman

**Affiliations:** 1 Gerodontology Research Group (GIOG), Department of Oral Rehabilitation, University of Talca, Talca, Chile; 2 Cariology Unit, Department of Oral Rehabilitation, University of Talca, Talca, Chile; 3 Interdisciplinary Excellence Research Program on Healthy Aging (PIEI-ES), University of Talca, Talca, Chile; 4 Institute of Mathematics and Physics, University of Talca, Talca, Chile

**Keywords:** Quality of life, Oral health, OHIP, Validation studies, Aging, Chile

## Abstract

**Background:**

The OHIP-49 is widely used to assess oral health-related quality of life, but its length makes it time-consuming and difficult to use. An abbreviated version of the OHIP-49 with fourteen items has been validated for older adults, but not in Chile. The aim was to develop and validate the Spanish version of the OHIP-14 in an elderly Chilean population.

**Methods:**

Two studies were conducted; a cross sectional to develop and a retrospective study to validate the OHIP-14Sp. The OHIP-49Sp was applied to 490 older adults and the fourteen questions with the higher impact on oral health-related quality of life were selected through linear regression. These items were applied on a retrospective dataset of 85 older adults to test internal consistency (Cronbach’s alpha). A discriminative validity analysis was performed along with the assessment of sociodemographic (age and gender, educational level) and clinical variables (number of teeth, caries lesions, CPITN, prosthetic needs and prosthetic functionality). Data were analyzed using Mann–Whitney U, Student’s t and one-way ANOVA tests with a 95% confidence level and finally were analyzed by a Multivariate Logistic Regression Model.

**Results:**

High internal consistency values were obtained for the OHIP-14Sp instrument (0.91). There was an association between the OHIP-14Sp scores and the presence of caries (p = 0.003), need for complex periodontal treatment (p = 0.002), prosthetic needs (p < 0.0001) and age younger than 70 years-old (p < 0.0001). Subjects with periodontal treatment need were more likely to report reduced oral health-related quality of life (PR = 2.10).

**Conclusions:**

The OHIP-14Sp proved to be a consistent and valid tool to assess oral health-related quality of life when tested in Chilean older adults.

## Background

The global accelerated increase in life expectancy demands activities intended to improve the quality of life of the elderly population [1]. Affecting functional, psychological and social aspects of daily life, poor oral health decidedly impacts on people’s quality of life [2]. To assess the extent at which oral health influences quality of life, multiple instruments have been developed, specifically in older adults. The Oral Health Impact Profile (OHIP) has been widely used in several countries to study oral health-related quality of life (OHRQoL). Originally comprising 49 questions, OHIP was intended for the self-report of dysfunction, discomfort and disability attributed to the oral conditions [3]. Lengthy tools, like the OHIP-49, may impose restrictions to be used in some settings, including extensive completion time, increased administration costs and data management, difficulties in the application to frail elderly adults and lower answer rates when compared to shorter versions, leading to a substantial data loss [4].

To optimize the application of the OHIP-49, Slade et al. developed a 14-question short version of the instrument [5], which has been validated in various languages, including Spanish [6-21]. To date, two validated versions of the OHIP-14 in the Spanish language have been published. One validation was performed in Spanish adults [15] and the other in an elderly Mexican population [18]. Since regional cultural and social differences and age group influence OHRQoL [22], the use of validated instruments in different languages and cultural contexts improves the orientation and values reflected by these measurements [23]. Although we have validated the complete version of the OHIP in Chile [24], an abbreviated OHIP-14 for older adults is not available in the country. The purpose of this study was, therefore, to develop an abbreviated OHIP with 14 questions and also to validate the new tool using an existent database, in an elderly Chilean population.

## Methods

### Sample group and data collection

Two different studies were conducted; a cross-sectional study to develop the abbreviated OHIP-14Sp with 490 community-dwelling older adults and a retrospective study to validate the instrument that used a previously obtained database of 85 community-dwelling older adults [24].

A convenience sample of patients was recruited from the School of Dentistry of the University of Talca, the Dental Specialties Clinic of the Talca’s Regional Hospital, social clubs for older adults and community healthcare centers from Talca. A sample size of 490 subjects was required, based on having 10 participants per question from the OHIP-49 [25,26]. In both studies, eligible participants should be community-dwelling and 60 years-old or older. Exclusion criteria were cognitive impairment and alcoholism. The study’s protocol was approved by the Bioethics Committee from the University of Talca. The study was conducted between June and October, 2012.

After receiving detailed information regarding the project and signing an Informed Consent Form, two trained dentists applied the OHIP-49Sp, previously validated in an elderly Chilean population by our group [24]. Each question was read one by one by one of the researchers, assuring the subject’s comprehension of each item. A printed chart with a Likert-type scale using clear and big letters was used in each question, as a visual reference to facilitate the answers. This ordinal scale has been shown to be valid as a response scale for this kind of survey [27]. After the subject’s answer, the researchers completed the questionnaire. This method was used due to high number of Chilean older adults with low educational levels and the high prevalence of visual problems.

By adding the final score of each of its seven domains, the final OHIP-49Sp score was obtained, ranging between 0 and 196 points. Using the OHIP-49Sp results, a linear regression model was done to obtain the abbreviated version of the instrument with 14 questions, as previously reported [5]. A controlled stepwise selection procedure was used to select 14 items that mainly contributed to R^2^ with the condition that only two items from each conceptual dimension were permitted to enter the regression model.

For the validation study, the already 14 selected questions from the cross-sectional study were tested against a retrospective cohort of 85 subjects that had been previously assessed during the validation of the Spanish version of the OHIP-49 [24]. Internal consistency was determined using the Cronbach’s α test as well as inter-item and item-total correlations. Scores were calculated using the additive method, considering values between 0 and 56, which has demonstrated excellent discriminatory ability [11,28,29].

### Discriminant validity

The 14 selected questions in the cross-sectional study were contrasted with sociodemographic and clinical variables obtained in the retrospective study of 85 subjects: Number of teeth (20–28, 10–19, 1–9 or edentate); caries lesions (Yes/No) according to WHO criteria [30]; Community Periodontal Index of Treatment Needs (CPITN) [31]; Prosthetic Need and Functional Assessment of Dentures (FAD) [32]. Parametric one-way ANOVA and non-parametric Kruskal-Wallis tests were used.

### Multivariate logistic regression analysis

Data were analyzed through frequency distribution and bivariate analysis with Fisher’s Exact Test. The dependent variable was the OHIP-14Sp, which was dichotomized using two categories as cutting point: never or hardly ever and occasionally, fairly often or very often. Independent variables were the abovementioned sociodemographic and clinical. Only factors identified by Fisher’s Exact Test were used in the Multivariate Logistic Regression Model. The odds ratios (ORs) were calculated to determine the strength of the association between the dependent and independent variables. Statistical analyses were performed using the SPSS v15.0 software (IBM Corporation, Somers, NY, USA).

## Results

Of the 490 subjects assessed previously with the OHIP-49Sp, 68.97% were female and 31.03% were male, with a mean age of 67.02 ± 5.81 years old.

When analyzing the OHIP-49Sp scores, the highest mean and median were 11.06 and 10.0, respectively. The dimension of the instrument that showed more negative impact of oral health on quality of life was Functional Limitation. The lowest impact in quality of life was found in the Social Disability (mean 1.73 and median 0) and Handicap (mean 2.76 and median 0) domains (Table 1).

**Table 1:** The OHIP-49 Sp scores for each domain in the population of the studied older adults

**Domain**	**Maximum possible**	**Minimum observed**	**Maximum observed**	**Mean**	**SD**	**Median**
Functional Limitation	36	0	32	11.06	7.15	10
Physical Pain	36	0	26	8.31	6.15	8
Psychological Discomfort	20	0	20	6.24	5.6	5
Physical Disability	36	0	30	7.2	6.96	5
Psychological Disability	24	0	23	3.73	4.99	2
Social Disability	20	0	18	1.73	3.13	0
Handicap	24	0	24	2.76	4.17	0
Global	196	0	154	40.95	31.42	34

To obtain the selected questions of the OHIP-14Sp, a linear regression model was carried out with the data previously obtained from the OHIP-49Sp. Thus, only the two questions from each dimension that more significantly contributed to the adjusted R^2^ were selected. Only two questions from Slade’s original OHIP-14 matched the present OHIP-14Sp: Q43 “difficulty doing jobs” and Q48 “unable to function”, corresponding to Social Disability and Handicap domains, respectively (Table 2). The most negative impact on the OHIP-14Sp arose from the Functional Limitation and Psychological Discomfort domains. Specifically, in the questions “breath stale” (46%) and “felt uncomfortable about the appearance” (39%). The negative impacts in the Psychological Disability, Social Disability and Handicap domains was very low (questions ranging from 10 to 11%) (Table 2).

**Table 2:** Regression analysis of the OHIP-49 items and prevalence of negative impact among subjects

**Domains and items**	^**a**^**R**^**2 **^**for OHIP-14Sp**	^**s**^**R**^**2 **^**for OHIP-14 (Slade)**	**% Subjects with negative impact**
OHIP-49Sp			
Functional limitation			
Q1: difficulty chewing	*	*	0.54
Q2: trouble pronouncing words	*	0.016	0.35
Q3: noticed tooth that doesn’t look right	*	*	0.48
Q4: appearance affected	*	*	0.42
Q5: breath stale	0.05	*	0.46
Q6: taste worse	*	0.032	0.43
Q7: food catching	*	*	0.75
Q8: digestion worse	0.10	*	0.19
Q9: dentures not fitting	*	*	0.40
Physical pain			
Q10: painful aching	*	0.024	0.37
Q11: sore jaw	*	*	0.28
Q12: headaches	*	*	0.08
Q13: sensitive teeth	0.14	*	0.33
Q14: toothache	0.05	*	0.35
Q15: painful gums	*	*	0.47
Q16: uncomfortable to eat	*	0.14	0.59
Q17: sore spots	*	*	0.46
Q18: uncomfortable dentures	*	*	0.39
Psychological discomfort			
Q19: worried by dental problems	*	*	0.68
Q20: self-conscious	*	0.057	0.41
Q21: dental problems made you miserable	0.03	*	0.33
Q22: felt uncomfortable about the appearance	0.03	*	0.39
Q23: felt tense	*	0.56	0.36
Physical disability			
Q24: speech unclear	0.04	*	0.34
Q25: others misunderstood	0.06	*	0.21
Q26: less flavor in food	*	*	0.43
Q27: unable to brush teeth	*	*	0.25
Q28: avoid eating	*	*	0.47
Q29: diet unsatisfactory	*	0.003	0.22
Q30: unable to eat (dentures)	*	*	0.25
Q31: avoid smiling	*	*	0.33
Q32: interrupt meals	*	0.004	0.30
Psychological disability			
Q33: sleep interrupted	0.11	*	0.10
Q34: upset	0.05	*	0.26
Q35: difficult to relax	*	0.087	0.27
Q36: depressed	*	*	0.27
Q37: concentration affected	*	*	0.20
Q38: been embarrassed	*	0.006	0.39
Social disability			
Q39: avoid going out	*	*	0.16
Q40: less tolerant of others	0.03	*	0.11
Q41: trouble getting on with others	*	*	0.20
Q42: irritable with others	*	0.002	0.12
Q43: difficulty doing jobs	0.09	0.007	0.11
Handicap			
Q44: your general health has worsened	*	*	0.21
Q45: financial loss	*	*	0.15
Q46: unable to enjoy people’s company	*	*	0.20
Q47: life unsatisfying	*	0.011	0.28
Q48: unable to function	0.06	0.001	0.11
Q49: unable to work	0.02	*	0.11

^a^Items selected for the OHIP-14Sp.

^s^Items of the original OHIP-14 developed by Slade.

*:Data not shown. Only R^2^ scores for questions included in the OHIP-14 are presented.

Once obtained the OHIP-14Sp, internal consistency was assessed using the OHIP-49Sp validation database with 85 subjects [24]. The sample was 61.18% female and 38.82% male, with an average age of 69.02 ± 7.82 years; 32.94% of the participants had between 20 and 28 teeth and 27.06% were edentulous. Among subjects having at least one tooth, 52.9% had at least one carious lesion and 100% required periodontal treatment, either simple or complex (CPITN 2 or 3). Prosthetic needs for either complete or removable partial denture was 74.12% and between those already wearing one, 65.5% reported poor functionality.

By analyzing the matrix of inter-item correlations (Table 3), a positive correlation between all items was found, ranging from 0.23 (the relationship between “breath stale” and “unable to work”) to 0.81 (the relationship between “speech unclear” and “others misunderstood”). The homogeneity of the scale was evaluated on the basis of the corrected item-total correlation coefficients. These analyses consider the correlation between each individual item in the scale and the rest of the scale with the item of interest eliminated. The corrected item-total correlation coefficients ranged from 0.42 to 0.75. All values were above the minimum corrected item-total correlation of 0.20, which has been recommended for the inclusion of an item in a scale [33]. Cronbach’s α for the total instrument total was 0.91 (Table 4).

**Table 3:** Reliability analysis based on the OHIP-14Sp inter-item correlation

**Impact item**	**1**	**2**	**3**	**4**	**5**	**6**	**7**	**8**	**9**	**10**	**11**	**12**	**13**	**14**
1 Breath stale	1.00													
2 Digestion worse	0.37	1.00												
3 Sensitive teeth	0.39	0.36	1.00											
4 Toothache	0.39	0.40	0.55	1.00										
5 Dental problems made you miserable	0.33	0.45	0.27	0.46	1.00									
6 Felt uncomfortable about the appearance	0.48	0.52	0.30	0.32	0.63	1.00								
7 Speech unclear	0.32	0.52	0.17	0.33	0.55	0.58	1.00							
8 Others misunderstood	0.38	0.53	0.27	0.37	0.59	0.66	0.81	1.00						
9 Sleep interrupted	0.33	0.44	0.26	0.37	0.33	0.42	0.24	0.34	1.00					
10 Upset	0.37	0.42	0.29	0.28	0.58	0.66	0.44	0.53	0.62	1.00				
11 Less tolerant of others	0.44	0.32	0.30	0.28	0.37	0.37	0.32	0.41	0.41	0.36	1.00			
12 Difficulty doing jobs	0.31	0.41	0.05	0.10	0.45	0.46	0.57	0.55	0.35	0.46	0.41	1.00		
13 Unable function	0.38	0.52	0.24	0.35	0.55	0.56	0.46	0.55	0.55	0.56	0.37	0.53	1.00	
14 Unable to work	0.23	0.42	0.25	0.27	0.37	0.41	0.40	0.44	0.60	0.51	0.39	0.44	0.56	1.0

**Table 4:** Reliability analysis based on the corrected item-total correlation and Cronbach’s alpha coefficient if item deleted

**Impact item**	**Corrected item-total correlation**	**Cronbach’s Alfa if item deleted**
1 Breath stale	0.52	0.90
2 Digestion worse	0.62	0.90
3 Sensitive teeth	0.42	0.91
4 Toothache	0.50	0.90
5 Dental problems made you miserable	0.72	0.89
6 Felt uncomfortable about the appearance	0.75	0.89
7 Speech unclear	0.62	0.90
8 Others misunderstood	0.72	0.90
9 Sleep interrupted	0.58	0.90
10 Upset	0.72	0.89
11 Less tolerant of others	0.52	0.90
12 Difficulty doing jobs	0.50	0.90
13 Unable function	0.75	0.89
14 Unable to work	0.57	0.90
**Global**	**0.91**	

Results from the discriminative validity tests by number of teeth showed that the highest mean scores for the OHIP (22.93 ± 13.16) and for each domain, were observed in subjects with 1–9 teeth. Despite the latter, the global OHIP-14Sp scores showed no significant relationship with number of remaining teeth (Table 5). A statistically significant association was found between the global OHIP-14Sp score and caries (p = 0.003). Similarly, a significant association was observed between caries and Functional Limitation, Psychological Discomfort and Physical Disability domains (Table 5). Regarding the Community Periodontal Index of Treatment Needs (CPITN), all subjects had Simple (TN2) or Complex Periodontal Treatment Needs (TN3). The highest mean and median values for the global OHIP and all domains (except Physical Pain) were found between subjects having Complex Periodontal Treatment needs. A statistically significant association was observed between CPITN and the global OHIP-14Sp score (p = 0.002) and all domains, except Physical Pain (Table 5).

**Table 5:** Discriminant validity of the OHIP-14Sp based on the oral variables of the subjects

	**Domain**							
**Variable**	**Functional limitation**	**Physical pain**	**Psychological discomfort**	**Physical disability**	**Psychological disability**	**Social disability**	**Handicap**	**Global OHIP-14 Sp**
	**Number of teeth**							
**20 to 28** (n=28)	2.36 (1.85) 2	2.39 (1.75) 2	3.32 (2.75) 3	1.54 (2.35) 0	2.25 (2.38) 2	1.39 (1.75) 0	1.32 (2.33) 0	14.57 (11.67) 12.5
**10 to 19** (n=19)	2.74 (2.83) 2	2.89 (2.58) 2	3.21 (3.08) 2	1.21 (1.90) 0	2.84 (2.36) 3	1.11 (1.45) 0	1.16 (1.68) 0	15.16 (11.38) 12
**1 to 9** (n = 15)	3.80 (2.91) 4	3.40 (1.99) 4	5.13 (2.85) 6	3.67 (2.23) 4	2.80 (2.11) 2	1.87 (2.03) 1	2.27 (2.60) 1	22.93 (13.16) 23
**Edentate** (n = 23)	2.17 (1.85) 2	1.52 (1.90) 0	3.04 (2.25) 2	1.96 (2.29) 1	2.35 (2.19) 2	1.13 (1.63) 0	1.13 (1.98) 0	13.30 (9.63) 12
p	0.3	0.03	0.1	0.01	0.7	0.5	0.3	0.08
	**Caries Lesions**							
**Yes** (n = 45)	3.42 (2.28) 4	3.04 (2.11) 3	4.18 (2.90) 5	6.29 (2.49) 2	2.89 (2.38) 2	1.64 (1.64) 2	1.73 (2.25) 1	19.36 (12.14) 18
**No** (n = 17)	1.78 (2.09) 1	1.78 (1.93) 1	2.83 (2.47) 2	1.40 (2.04) 0	2.08 (2.06) 2	1 (1.72) 0	1.03 (2.02) 0	11.88 (10.92) 10.50
p	0.0009	0.005	0.02	0.03	0.09	0.08	0.13	0.003
	**CPITN**							
**TN2** (n = 31)	2.10 (2.23) 2	2.35 (2.24) 2	2.90 (2.71) 2	1.26 (2.11) 0	1.97 (2.23) 2	0.77 (1.38) 0	0.65 (1.50) 0	12 (11.21) 9
**TN3** (n = 31)	3.55 (2.54) 4	3.23 (1.87) 3	4.55 (2.98) 5	2.65 (2.44) 3	3.16 (2.24) 3	2.06 (1.82) 2	2.35 (2.52) 2	21.55 (12.77) 23
p	0.01	0.10	0.02	0.02	0.03	0.002	0.002	0.002
	**Prosthetic Need**							
**No** (n = 22)	1.23 (1.69) 0	2.05 (1.70) 2	1.91 (2.09) 2	0.36 (1.05) 0	1.18 (1.37) 0.5	0.64 (1.14) 0	0.23 (0.61) 0	7.59 (6.26) 6
**Yes** (n = 63)	3.14 (2.33) 3	2.59 (2.23) 2	4.11 (2.77) 4	2.51 (2.41) 2	2.97 (2.33) 2	1.59 (1.80) 1	1.81 (2.35) 1	18.71 (12.37) 16
p	0.0007	0.3	0.001	<0.0001	<0.0001	0.005	<0.0001	<0.0001

Mean, (SD), median.

Older adults having Prosthetic needs presented the highest scores in all domains. Indeed, a very strong association (p < 0,0001) was observed between the Physical Disability, Psychological Disability, Handicap and the global OHIP-14Sp. Likewise, a significant association was detected between the other domains and Prosthetic needs, except with Physical Pain (Table 5). When Denture Functionality was evaluated, no statistically significant association was observed for Upper or Lower Functionality and the OHIP-14Sp scores, except for Upper Functionality and the Functional Limitation dimension. Nonetheless, highest scores for the global OHIP-14Sp and all domains were found in subjects whose Denture Functionality was low (Table 5).

When age was considered, highest OHIP-14Sp scores were observed in the youngest group (60 to 69 years). A very strong association (p < 0.0001) was seen between Psychological Disability, Social Disability and the Handicap domains and the global OHIP-14Sp score. A significant association was also found between the OHIP-14Sp and the other domains, except for Physical Pain and Physical Disability domains (p > 0.05) (Table 6).

**Table 6:** Discriminant validity of the OHIP-14Sp based on the age of the subjects

	**Domain**							
**Age**	**Functional Limitation**	**Physical Pain**	**Psychological Discomfort**	**Physical Disability**	**Psychological Disability**	**Social Disability**	**Handicap**	**OHIP-49 Sp**
60 to 69 (n = 52)	3.23 (2.38) 3.5	2.73 (2.36) 3	4.23 (2.89) 5	2.27 (2.53) 1	3.17 (2.43) 3	1.85 (1.82) 2	2.02 (2.49) 1	19.5 (13.22) 16
70 and + (n = 33)	1.73 (1.96) 1	2 (1.58) 2	2.45 (2.21) 2	1.45 (1.92) 1	1.45 (1.44) 2	0.55 (1.12) 0	0.42 (0.87) 0	10.06 (7.11) 10
p	0.003	0.09	0.003	0.1	<0.0001	<0.0001	<0.0001	<0.0001

Mean, (SD), median.

When the bivariate analysis was performed using Fisher’s Exact Test, the OHIP-14Sp score was significantly associated with caries (p = 0.004), CPITN (p = 0.010), number of teeth (p = 0.025) and educational level (p = 0.040). Then, all independent variables found significant by the test were used into a one-step multivariate Logistic Regression Model, along with the dichotomized OHIP-14Sp variable as the dependent variable. Two models were tested: one for participants with teeth and a second for those edentulous. Subjects with periodontal treatment needs (CPITN 2–3) were more likely to report reduced OHRQoL (PR = 2.10).

## Discussion

Results from this study validate the Spanish version of the OHIP-14 as a valid instrument to assess OHRQoL in elderly Chileans. Herein, clinical variables were similar to those used in other studies [8,9,18]. Nevertheless, we decided to additionally include Prosthetic Functionality, as another variable in the analysis [32], mainly due to the high tooth loss rates of Chilean elderly population [34]. In the present study, tooth loss was high with 17.65% of all subjects having 1–9 remaining teeth and 27.06% totally edentulous.

If the low educational level of the current cohorts of elderly Chileans is taken into consideration, some reading issues with the questionnaire may have been possible. To that effect, the OHIP-14Sp was administered by trained interviewers, so bias from misunderstanding was avoided. This procedure has been previously used [3,8-10,35], with the main advantage that it ensures a 100% response rate and a good comprehension for all items, when compared with a self-administered survey. Yet, it has been reported that the psychometric properties of the original English language OHIP-14 were not related with the administration method (self-administered versus interview) [36].

Most the OHIP-14 versions have been based only on the translation and linguistic adaptation of the original English language questionnaire [9,11,12,14-16,19,20,37]. Like in the original study [5], the method to obtain the OHIP-14Sp here was a linear regression with two questions per dimension. This item-selection method has demonstrated to be more satisfactory than the internal reliability analysis or factor analysis [4]. Although it has been used in similar studies in Taiwan, China and Korea [6,10,13], few coincidences occurred between questions selected on these studies and the Chilean version. Most matches occurred with the Chinese version, in four questions. These results support the fact that instrument validation must be performed according to the cultural context of the population to be analyzed. In fact, cultural and social differences may lead to important discrepancies in the results obtained with the instruments.

Besides Slade’s methodology used to obtain a shortened OHIP version, Locker et al. [4] developed a different strategy, based on the modified clinical impact method suggested originally by Juniper et al., [38]. Both methodologies were applied in the development of the German short forms of the OHIP, resulting equally satisfying. According to the authors, the short OHIP questionnaires maintain the idea of the OHRQoL as a construct, regardless of the methodologies used [17].

Despite obtaining a low internal consistency in some domains, a high internal consistency was demonstrated by the global instrument (Cronbach’s α = 0.91). The global consistency found here was higher than the originally reported by Slade and Spencer [5] and other authors [6,9,10,12,14,15,20,39]. Yet, our results are in consistency with other studies [7,11,16,18,37]. Internal consistency of the OHIP-14Sp was lower than that obtained in a previous OHIP-49Sp validation [24], but it should not be interpreted as a pitfall, as alpha values are influenced by the length of the questionnaire [40]. Still, Cronbach’s α coefficient values higher than 0.6 indicate that the instrument has a high internal consistency [41]. The high alpha value (0.91) indicates that the 14 items of the OHIP-14Sp scale accurately measure the same construct. In that sense, OHRQoL can be assessed at the item level; per dimension or as a construct. Although limited in the case of shortened versions, choosing to characterize OHRQoL per item it may provide useful information. It is known that shortened versions of the OHIP do not describe domains accurately. Hence, it is suggested that analyses of the results obtained from these instruments should not be considered by domains separately, considering that they are represented by only two questions. The oversimplification may lead to negative interpretations of the results of the test [42]. Still, saving resources and time through the application of shortened instruments should be evaluated considering these disadvantages. Therefore, the use of these instruments should be considered only when the main purpose is the characterization of the construct OHRQoL [17]. Indeed, OHRQoL for adults and older adults has been considered only as a construct [43,44].

The single or multidimensionality of the OHIP-14 has been widely debated [45-48]. The variability of this findings is due to several influences such as vast differences in the studied populations, OHIP forms of varying lengths were used in each study and small samples sizes. Recent studies report that despite the OHIP data can be well characterized by a unidimensional model, the four-dimensional (Oral Function, Oro-facial Pain, Oro-facial Appearance and Psychosocial Impact) may fit even better than the unidimensional model [49,50]. Thus, in the present study, all analyses were performed using global scores of the instruments, which had an excellent internal consistency.

The corrected item-total correlation coefficients ranged from 0.42 to 0.75 and were above the minimum level of 0.20, which has been recommended for including an item in a scale. These scores are similar to those found in other studies [7,12,16,18], which indicates good homogeneity and prevents the elimination of some items from the OHIP-14Sp.

In our study, Functional Limitation and Psychological Discomfort dimension showed a higher prevalence of negative impact, as reported in previous studies [5,19]. Also, the least negative impact was in the Psichological Disability, Social Disability and Handicap domains, which is similar to other studies [5,19]. Our results provide additional support to Locker’s theoretical model of oral health, indicating that Social Disability and Handicap are less frequent and measure the most comprehensive impact on quality of life [51].

Unlike the OHIP-49Sp [45], discriminative validation was not related with number of teeth in the OHIP-14Sp. It is expected that a more frequent use of dentures would minimize the consequences of tooth loss [6]. Reasons for these findings may arise from the fact that older adults with few teeth will probably wore dentures. So, even if they were not functional, self-perception of quality of life was better explained by having an esthetically acceptable prosthesis. Differences with other studies may derive from question interpretation or cultural characteristics of the studied population [52].

When evaluating discriminative validity based on clinical variables (Table 5), the most robust associations were observed between the presence of carious lesions, CPITN and Prosthetic needs, the latter with similar results with other studies [52].

Regarding age, older individuals presented a lower negative impact of the oral diseases in their quality of life than youngest individuals. The inverse relation between age and the OHIP scores has been reported in other studies [29,52,53]. The theory of response shift may explain why community dwelling seniors, and the elderly population in general, may report fewer impact in certain domains [54]. With aging, subjects tend to accept the fact that health deteriorates, and therefore, oral problems are considered less significant [55]. A recent study analyzed the paradox of the better subjective oral health perception in older age. The findings showed that experience of oral disease is more deleterious to subjective oral health when it occurs early in adulthood than when it occurs in old age, likely reflecting high expectations of young generations and, conversely, great resilience in the oldest generation [52].

Even though Fisher’s Exact Test showed significant associations between several variables and the OHIP-Sp scores, only few remained significant when entered into the logistic regression model. From the analysis, only CPITN resulted in a statistically significant increase in the risk of oral disease. Those who had Complex Periodontal Treatment Need had 2.1 times more risk of having a negative perception of quality of life. This can be justified due to the fact that advanced stages of periodontal disease involve recessions, hypersensitivity, tooth mobility, pain, swelling, bleeding, aesthetic and phonetic impairment, among others [56]. These collateral effects from advanced periodontal disease may explain the association between poor quality of life and complex periodontal treatment needs found in the present study.

We deem important to acknowledge that for the discriminative validity analysis, subjects from the validation of the OHIP-49Sp were considered. Thus, we did not consider a specific sample size for the OHIP-14Sp. Sample size was estimated only for the OHIP-49Sp validation [24]. Also important, the female to male ratio was 70% to 30%, respectively. The population in the present study was obtained by convenience from various sources and thus, the proportion not necessarily represents the actual ratio between females and males. Yet, expected projections indicate that women will outnumber men by 2020 in about 30%, due to higher life expectance of women [57]. The differences could also be explained by the fact that more women were prone to participate is expected in this type of study with older adults. On the other hand, the cross-sectional design, although well documented and its discriminative validity being established [53,58], reduces the level of evidence on the associations found. We suggest implementing longitudinal studies to increase the degree of evidence and assess the sensitivity of the OHIP-14Sp to detect changes in OHRQoL perception after therapeutic interventions.

## Conclusions

This study confirmed that the OHIP-14Sp is a valid instrument when used in Chilean older adults. We expect that the OHIP-14Sp will be a useful alternative to the OHIP-49Sp when time and resources are limited. Further, the OHIP-14Sp allows gathering data to be used and compared national and internationally.

## Competing interests

The authors declare that they have no competing interests.

## Authors’ contribution

SL conceived the research ideas, organized the clinical design, collected clinical data and drafted the first manuscript. DB collected clinical data, revised the initial draft and discussed the results. GC performed the statistical analyses and helped with the discussion. RAG discussed the data, edited and prepared the final version of the manuscript. All authors read the final version of the manuscript.

## Authors’ information

Soraya León is currently a PhD student at the Faculty of Dentistry of the Federal University of Rio Grande do Sul, Porto Alegre, Brazil.

## Pre-publication history

The pre-publication history for this paper can be accessed here:

http://www.biomedcentral.com/1472-6831/14/95/prepub
